# Aloe-emodin inhibits nasopharyngeal carcinoma by modulating telomerase activity involving the c-Myc/E2F1 axis

**DOI:** 10.3389/fphar.2026.1850685

**Published:** 2026-07-20

**Authors:** Mengqin Li, Chunhong Li, Xin Zeng, Jiahua Hu, Han Su, Jiayi Huang, Min He, Jianhong Tang

**Affiliations:** 1 The Second Affiliated Hospital of Guilin Medical University, Guilin, Guangxi, China; 2 College of Pharmacy, Guilin Medical University, Guilin, Guangxi, China; 3 Guangxi Key Laboratory of Diabetic Systems Medicine, Guilin Medical University, Guilin, Guangxi, China; 4 Guangxi Health Commission Key Laboratory of Glucose and Lipid Metabolism Disorders, The Second Affiliated Hospital of Guilin Medical University, Guilin, Guangxi, China; 5 The First Affiliated Hospital of Guilin Medical University, Guilin, Guangxi, China; 6 Department of Pharmacy, The Second Affiliated Hospital of Guilin Medical University, Guilin, Guangxi, China; 7 School of General Medicine, Guilin Medical University, Guilin, Guangxi, China

**Keywords:** aloe-emodin, bioinformatics, hTERT, NPC, telomerase

## Abstract

**Background:**

The telomerase catalytic subunit hTERT is a critical oncogenic driver in nasopharyngeal carcinoma (NPC). The specific role of aloe-emodin (AE) in regulating hTERT in NPC has not been systematically investigated. The regulatory network involving hTERT and its upstream transcription factors, c-Myc and E2F1, offers a promising therapeutic avenue.

**Methods:**

Bioinformatics was used to analyze hTERT expression and its prognostic significance in head and neck squamous cell carcinoma and nasopharyngeal carcinoma. Molecular docking simulations were conducted to investigate the potential interaction between AE and hTERT. hTERT expression was assessed in normal nasal epithelial cells and NPC cell lines. Effects of AE on hTERT, c-Myc, and E2F1 were measured using qRT-PCR and Western blotting. Telomerase activity was evaluated through TRAP-qPCR and PAGE. hTERT was knocked down using siRNA. Cell proliferation, migration, and invasion were analyzed using CCK-8, EdU, colony formation, and Transwell assays. ROS levels were quantified using DCFH-DA staining. *In vivo*, a subcutaneous xenograft model was established to assess AE’s anti-tumor efficacy alone and with shRNA-mediated hTERT knockdown. Organ toxicity was evaluated by H&E staining. Tumor tissues were analyzed by immunohistochemistry for hTERT, c-Myc, E2F1, Ki-67, and Cleaved Caspase-3.

**Results:**

Bioinformatics analysis revealed elevated hTERT expression in HNSC tissues and NPC cell lines Molecular docking predicted a potential interaction between AE and the catalytic pocket of hTERT. *In vitro*, AE treatment decreased c-Myc, increased E2F1, and reduced hTERT transcription. AE also reduced telomerase activity, increased ROS levels, and suppressed proliferation, migration, and invasion in NPC cells. *In vivo*, AE showed anti-tumor activity. Combined with hTERT knockdown, AE produced synergistic tumor suppression and enhanced modulation of the c-Myc/E2F1/hTERT axis and oxidative stress. Immunohistochemistry revealed suppressed proliferation and increased apoptosis in tumor tissues of the combination group. No significant organ toxicity was observed.

**Conclusion:**

This study presents the first systematic validation of AE’s anti-NPC effects. These include hTERT suppression, modulation of the c-Myc/E2F1 network, and induction of oxidative stress. These findings suggest a potential therapeutic target and strategy for NPC. The results support the clinical translation of AE.

## 1 Introduction

Nasopharyngeal carcinoma (NPC) is geographically restricted, with the highest incidence in East and Southeast Asia (Jainar et al., 2025). China accounts for a large share of new NPC cases worldwide. NPC ranks first among head and neck cancers in China (Qian et al., 2025). Due to its occult location, most patients are diagnosed at advanced stages. These advanced stages are associated with high metastatic potential (Hou et al., 2026). Although radiotherapy and chemotherapy have advanced, challenges persist. About 10%–20% of patients experience recurrence or metastasis, with a median post-recurrence survival of just 10–15 months (Chen et al., 2021). In addition, radiotherapy-related toxicities and chemotherapy resistance severely impact quality of life and clinical outcomes (Chen et al., 2021; Kamara et al., 2024). Immunotherapies show promise but bring significant adverse events. This underscores the urgent need for novel, low-toxicity therapies (Zhang et al., 2021).

Natural compounds are receiving significant attention for cancer therapy. Aloe-emodin (AE) is an anthraquinone derivative from Aloe vera, known for a wide range of pharmacological activities. Its antitumor effects across several malignancies stand out (Şeker Karatoprak et al., 2022). Mechanistically, AE induces autophagy in lung cancer via MAPK and AKT/mTOR signaling pathways. It has anti-proliferative effects in hepatocellular carcinoma through the PI3K/AKT pathway. It also inhibits breast cancer by suppressing hTERT and telomerase activity (Shen et al., 2020; Wang et al., 2020). Our previous studies show that AE suppresses proliferation and migration and induces apoptosis in NPC cells. However, the underlying molecular mechanisms are still unclear (Chen et al., 2023; He et al., 2025).

Telomerase is a well-established malignancy marker, and its catalytic subunit hTERT is highly expressed in NPC (Zhang et al., 2022; Roake and Artandi, 2020). Studies have confirmed that telomerase activity can serve as a diagnostic marker for detecting recurrent NPC cells. hTERT, the rate-limiting component of telomerase, is positively regulated by c-Myc, which directly activates hTERT transcription, thereby enhancing telomerase activity (Feijing et al., 2025; Saraswati et al., 2019). Conversely, studies have shown that E2F1 can bind the hTERT promoter and repress its expression in several cell lines (Won et al., 2004). Notably, elevated hTERT expression levels in the plasma of NPC patients are closely associated with tumor stage, tumor size, and lymph node metastasis (Fu et al., 2017). Beyond its canonical role in telomere maintenance, hTERT also regulates redox homeostasis: hTERT overexpression protects against ROS, whereas hTERT inhibition promotes ROS-induced apoptosis (Fu et al., 2017; Ait-Aissa et al., 2022; Jayaprasad et al., 2024; Liu et al., 2024).

Whether AE regulates the hTERT/c-Myc/E2F1 axis in NPC, and whether hTERT knockdown synergizes with AE via ROS, remains unknown, particularly *in vivo*. We hypothesize that AE inhibits NPC by modulating hTERT and its upstream regulators c-Myc and E2F1, thereby reducing telomerase activity and increasing ROS accumulation. To test this hypothesis, we focused on two key transcription factors that govern hTERT: c-Myc activates hTERT transcription, whereas E2F1 has been reported to repress it under certain cellular stress conditions (Won et al., 2004). However, whether AE modulates E2F1 to directly suppress hTERT in NPC, or whether the observed E2F1 upregulation merely reflects a secondary stress response, is unknown. We therefore combined bioinformatics, molecular docking, *in vitro* and *in vivo* experiments, and hTERT knockdown to elucidate the AE-hTERT regulatory axis and assess its therapeutic potential. To our knowledge, this is the first comprehensive investigation of the AE-hTERT regulatory axis in nasopharyngeal carcinoma, as the effects of AE on hTERT and on modulation of c-Myc/E2F1 have not been previously reported in this context.

## 2 Materials and methods

### 2.1 Materials

Human nasal epithelial cells (HNEpC) were purchased from Suyan Biological Technology Co., Ltd (Guangzhou, China). NPC cell lines CNE1, 5–8F, HONE1, and C666-1 were obtained from the Cell Center of Xiangya School of Medicine, Central South University, and Shanghai Yuchi Biotechnology Co., Ltd (Shanghai, China). AE was purchased from Shanghai Yuanye Biotechnology Co., Ltd (Shanghai, China). RPMI 1640 medium and EMEM medium were sourced from Gibco (United States) and Suyan Biological Technology Co., Ltd (Guangzhou, China), respectively. Fetal bovine serum (FBS) and penicillin-streptomycin solution were purchased from Vazyme Biotech (Nanjing, China) and Beyotime Biotechnology (Shanghai, China). TRIzol reagent and SYBR Green Master Mix were obtained from Jinsha Biotechnology Co., Ltd (Beijing, China) and Vazyme Biotech (Nanjing, China), respectively. The FastQuant cDNA First-Strand Synthesis Kit was purchased from Jinsha Biotechnology Co., Ltd (Beijing, China). The hTERT siRNA duplex (sense: 5′-AGACCAGCACUUCCUCUAdTdT-3′; antisense: 5′-UAGAGGAAGUGCUUGGUCUdTdT-3′) and negative control siRNA were synthesized by Sangon Biotech (Shanghai, China). The shRNA lentiviral vector targeting hTERT (target sequence: 5′-GAC​ATG​GAG​AAC​AAG​CTG​TTT-3′) and packaging services were provided by Wuhan Painuo Biotechnology Co., Ltd (Wuhan, China). Primary antibodies against hTERT (Cat# HY-P81110), c-Myc (Cat# 67447-1-Ig), E2F1 (Cat# GB11571-100), Ki67 (Cat# GB111499-100), Cleaved Caspase-3 (Cat# GB11532-100), and β-actin (Cat# 66009-1-Ig) were purchased from MedChemExpress, Proteintech, and Servicebio. HRP-conjugated secondary antibodies and ECL chemiluminescence kit were obtained from Beyotime Biotechnology and Life-iLab Biotech (Shanghai, China). BALB/c nude mice (SPF grade, Production License No.: SCXK 2021-0002) were purchased from Hunan SJA Laboratory Animal Co., Ltd (Changsha, China). The full-length human c-Myc coding sequence (NCBI Reference Sequence: NM_002467.6) was synthesized and cloned into the pcDNA3.1 (+) vector by Sangon Biotech (Shanghai, China). The empty pcDNA3.1 (+) vector from the same supplier was used as a negative control. MG132 (MedChemExpress, United States, Cat# HY-13259) was stored at −20 °C and diluted to 10 μM with culture medium before use.

### 2.2 Cell culture

HNEpC were cultured in EMEM medium supplemented with 10% FBS and 1% penicillin-streptomycin. NPC cell lines CNE1, 5–8F, HONE1, and C666-1 were maintained in RPMI 1640 medium with the same supplements. All cells were incubated at 37 °C in a humidified atmosphere containing 5% CO_2_. Based on preliminary experiments and our previous study (Chen et al., 2023), AE was dissolved in DMSO and used at 20 μM for mechanistic studies, with additional concentrations (10, 20, 30 μM) applied for dose-response assays.

### 2.3 Bioinformatics analysis

Pan-cancer expression of hTERT mRNA was analyzed using the UCSC Xena database (https://xena.ucsc.edu/) and visualized via the Sangerbox platform (http://sangerbox.com) and TIMER 2.0 (http://timer.cistrome.org). Differential expression and prognostic significance in HNSC were assessed using ENCORI (http://starbase.sysu.edu.cn), UALCAN (http://ualcan.path.uab.edu), and GEPIA (http://gepia2.cancer-pku.cn) databases. Univariate Cox regression was performed via Sangerbox to evaluate the prognostic value of hTERT across pan-cancer. Immune cell infiltration was assessed using the Sangerbox platform, with Spearman’s rank correlation used to analyze associations between TERT expression and infiltration levels of six immune cell types.

### 2.4 Quantitative real-time PCR

Total RNA was extracted from cells using TRIzol reagent, and 1 μg of RNA was reverse transcribed into cDNA using the FastQuant cDNA First-Strand Synthesis Kit. qPCR was performed on a CFX96 system using SYBR Green Master Mix with the following primers: hTERT (forward: 5′-CGG​AAG​AGT​GTC​TGG​AGC​AA-3′; reverse: 5′-GGA​TGA​AGC​GGA​GTC​TGG​A-3′), c-Myc (forward: 5′-CGT​CCT​CGG​ATT​CTC​TGC​TCT​C-3′; reverse: 5′-TCC​TCA​TCT​TCT​TGT​TCC​TCC​TCA​G-3′), E2F1 (forward: 5′-ATG​TTT​TCC​TGT​GCC​CTG​AG-3′; reverse: 5′-ATC​TGT​GGT​GAG​GGA​TGA​GG-3′), and β-actin (forward: 5′-TGA​CAC​CTC​ACC​TCA​CCC​AC-3′; reverse: 5′-CAC​TGT​CTT​CCG​CAA​GTT​CAC-3′). Relative gene expression was calculated using the 2^−ΔΔCt^ method with β-actin as the internal control.

### 2.5 Western blotting

Total protein was extracted from cells using RIPA lysis buffer, and protein concentrations were determined by BCA assay. Equal amounts of protein were separated by SDS-PAGE and transferred onto PVDF membranes. Membranes were blocked with 5% non-fat milk or 5% BSA, then incubated overnight at 4 °C with primary antibodies against hTERT, c-Myc, E2F1, and β-actin. After washing, membranes were incubated with HRP-conjugated secondary antibodies. Protein bands were visualized using an ECL chemiluminescence kit and quantified by densitometry using ImageJ software. β-actin served as the loading control.

### 2.6 TRAP-qPCR for telomerase activity

Telomerase activity was assessed using the telomeric repeat amplification protocol (TRAP) combined with qPCR and gel electrophoresis. Cells were lysed in CHAPS lysis buffer, and the supernatant containing telomerase extract was collected. The extract was incubated with TS primer and dNTPs at 25 °C for 30 min to allow telomerase-mediated extension, followed by heat inactivation at 95 °C for 10 min. The extension products were amplified by qPCR using SYBR Green Master Mix with TS and ACX primers. Relative telomerase activity was calculated using the 2^−ΔCt^ method, where ΔCt = Ct (sample)−Ct (negative control). To verify amplification specificity, qPCR products were analyzed by non-denaturing polyacrylamide gel electrophoresis.

### 2.7 Cell transfection

NPC cells were seeded at a density of 2 × 10^5^ cells per well in 6-well plates and cultured until reaching 60% confluence. For each well, 100 pmol of hTERT siRNA or negative control siRNA was diluted in 125 μL of Opti-MEM, mixed with 4 μL of LipoRNAi™ transfection reagent, and incubated at room temperature for 20 min to form transfection complexes. The complexes were then added to cells and incubated for 48 h. Transfection efficiency was verified by qPCR prior to functional experiments.

### 2.8 Synergy analysis by the Chou-Talalay method

The synergistic interaction between AE treatment and hTERT silencing was quantitatively evaluated using the Chou-Talalay combination index (CI) method. CI values were calculated from cell inhibition rates; CI < 0.9 indicated synergism, 0.9–1.1 indicated an additive effect, and >1.1 indicated antagonism.

### 2.9 Assessment of malignant biological behaviors

To evaluate the effects of AE and hTERT knockdown on NPC cell malignant phenotypes, the following assays were performed. Cells were divided into five groups: Control, NC (negative control siRNA), siRNA (hTERT siRNA), AE (20 μM AE), and siRNA + AE (hTERT siRNA combined with 20 μM AE). All experiments were performed in triplicate.

Cell viability was assessed using the CCK-8 assay. Cells were seeded in 96-well plates at 1 × 10^4^ cells per well. After 48 h of treatment, 10 μL of CCK-8 solution was added, incubated for 2 h, and absorbance was measured at 450 nm.

Proliferation was evaluated by colony formation and EdU incorporation assays. For colony formation, cells were seeded at 200 cells per well in 6-well plates and cultured for 14 days. Colonies were fixed with 4% paraformaldehyde, stained with 0.2% crystal violet, and counted. For the EdU assay, cells were seeded in 24-well plates at 1 × 10^4^ cells per well, incubated with 10 μM EdU for 4 h, fixed, permeabilized, and stained using a Click reaction kit. Nuclei were counterstained with Hoechst 33342, and EdU-positive cells were visualized under a fluorescence microscope.

Migration and invasion were evaluated using wound-healing and Transwell assays. For wound-healing assays, cells were seeded at 2 × 10^5^ cells per well in 6-well plates. A scratch was created using a 200 μL pipette tip, and wound closure was observed after 48 h. For Transwell assays, cells were seeded in serum-free medium into upper chambers (5 × 10^3^ cells per well), with complete medium containing 10% FBS in the lower chamber as a chemoattractant. For invasion assays, upper chambers were pre-coated with Matrigel. After 24 h, cells on the lower surface were fixed, stained with 0.1% crystal violet, and counted. For multiple comparisons among the five treatment groups, one-way ANOVA followed by Tukey’s post-hoc test was applied.

### 2.10 Measurement of intracellular ROS levels

Intracellular reactive oxygen species (ROS) levels were detected using the DCFH-DA fluorescent probe. NPC cells were seeded in 6-well plates at 5 × 10^5^ cells per well and treated for 24 h under the following conditions: Control, NC (negative control siRNA), AE (20 μM AE), NAC + AE (pretreated with 5 mM NAC for 2 h followed by 20 μM AE), siRNA (hTERT siRNA), and siRNA + AE (hTERT siRNA combined with 20 μM AE). After treatment, cells were incubated with 10 μM DCFH-DA in serum-free medium at 37 °C for 30 min in the dark. Fluorescence was observed under a fluorescence microscope (excitation/emission: 488/525 nm), and fluorescence intensity was quantified using ImageJ software. All experiments were performed in triplicate.

### 2.11 Molecular docking

Molecular docking was performed to predict the binding mode between AE and hTERT. The structure of AE (PubChem CID: 10207) was optimized using Open Babel. The crystal structure of hTERT was obtained from the Protein Data Bank (PDB ID: 9SI0) (Balch et al., 2026). Water molecules and original ligands were removed, and hydrogen atoms were added using PyMOL. Docking simulations were performed using AutoDock Vina, with the grid box centered on the reported active site of hTERT. The resulting binding conformations were visualized using PyMOL to analyze key interactions, including hydrogen bonds and hydrophobic contacts.

### 2.12 Construction of stable hTERT knockdown cell line

To establish C666-1 cells with stable hTERT knockdown for *in vivo* experiments, lentiviral transduction followed by puromycin selection was performed. The shRNA lentiviral vector targeting hTERT (target sequence: 5′-GAC​ATG​GAG​AAC​AAG​CTG​TTT-3′) was used. C666-1 cells were seeded in 96-well plates at 5 × 10^3^ cells per well and transduced with lentivirus at the optimal multiplicity of infection (MOI) determined by preliminary experiments. After 48 h, cells were selected with 2 μg/mL puromycin for 10–14 days, with medium renewed every 2–3 days. Surviving monoclonal cell clusters were expanded to establish stable knockdown cell lines. Knockdown efficiency was confirmed by qPCR prior to *in vivo* experiments.

### 2.13 Subcutaneous xenograft model

Eighteen 4-week-old male BALB/c nude mice were randomly divided into three groups (n = 6 per group): NC (inoculated with C666-1 cells stably expressing empty vector), AE (inoculated with C666-1 cells and treated with AE), and shTERT + AE (inoculated with C666-1 cells stably knocked down for hTERT and treated with AE). Cells in logarithmic growth phase were harvested and resuspended in a 1:1 mixture of serum-free medium and Matrigel at a concentration of 1 × 10^7^ cells/mL, and 2 × 10^6^ cells were subcutaneously injected into the right armpit of each mouse. When the average tumor volume reached approximately 80 mm^3^, mice in the AE and shTERT + AE groups received daily intraperitoneal injections of AE (20 mg/kg) for 10 consecutive days, while the NC group received an equal volume of saline. Tumor volumes were measured every 2–3 days. Twenty-four hours after the final administration, mice were euthanized, and tumor tissues were excised, weighed, and processed for further analysis. All animal experiments were approved by the Animal Ethics Committee of Guilin Medical University (Approval No.: GLMU-IACUC-202510141).

### 2.14 H&E staining

Heart, liver, and kidney tissues collected from nude mice were fixed in 4% paraformaldehyde, embedded in paraffin, and sectioned at 4–5 μm thickness. Sections were deparaffinized in xylene, rehydrated through a graded ethanol series, and stained with hematoxylin for 5 min, followed by eosin for 2 min. After dehydration and clearing, sections were mounted with neutral balsam and observed under a light microscope.

### 2.15 Immunohistochemistry

Tumor tissues from nude mice were fixed in 4% paraformaldehyde, embedded in paraffin, and sectioned at 4–5 μm thickness. Sections were deparaffinized in xylene, rehydrated through a graded ethanol series, and subjected to antigen retrieval in sodium citrate buffer (pH 6.0) using microwave heating. Endogenous peroxidase activity was blocked with 3% hydrogen peroxide, and non-specific binding was reduced by blocking with 5% BSA. Sections were incubated overnight at 4 °C with primary antibodies against hTERT, c-Myc, E2F1, Ki67, and Cleaved Caspase-3. After washing, sections were incubated with HRP-conjugated secondary antibodies, and the signal was visualized with DAB. Nuclei were counterstained with hematoxylin. Stained sections were observed and imaged under a light microscope.

## 3 Results

### 3.1 hTERT is highly expressed in HNSC with distinct prognostic features and overexpressed in NPC cell lines

Analysis of TCGA and GTEx databases revealed that hTERT expression was significantly upregulated in head and neck squamous cell carcinoma (HNSC; the TCGA-designated abbreviation for HNSCC) tumor tissues compared with normal tissue samples, as confirmed by ENCORI (520 tumor vs. 44 normal) and UALCAN (502 tumor vs. 44 normal) databases (Supplementary Figures S1A,B). Interestingly, survival analysis showed that HNSC patients with high hTERT expression had significantly longer overall survival than those with low expression (Supplementary Figures S1C,D; GEPIA: log-rank P = 0.034, HR = 0.75; UALCAN: P = 0.015), a pattern opposite to the typical pan-cancer prognostic trend.

Pan-cancer expression and survival analyses, as well as immune infiltration correlations, are provided in Supplementary Figures S2–S4. We further examined hTERT expression in two NPC-specific GEO datasets (GSE53819 and GSE12452). As shown in Supplementary Table S2, hTERT consistently showed positive log_2_FC values across all probes (range +0.019 to +0.136). However, no statistically significant differences were detected in any of the three analyses, likely due to the limited sample size (e.g., normal control n = 10 in GSE12452) and biological heterogeneity of clinical specimens.

**FIGURE 1:**
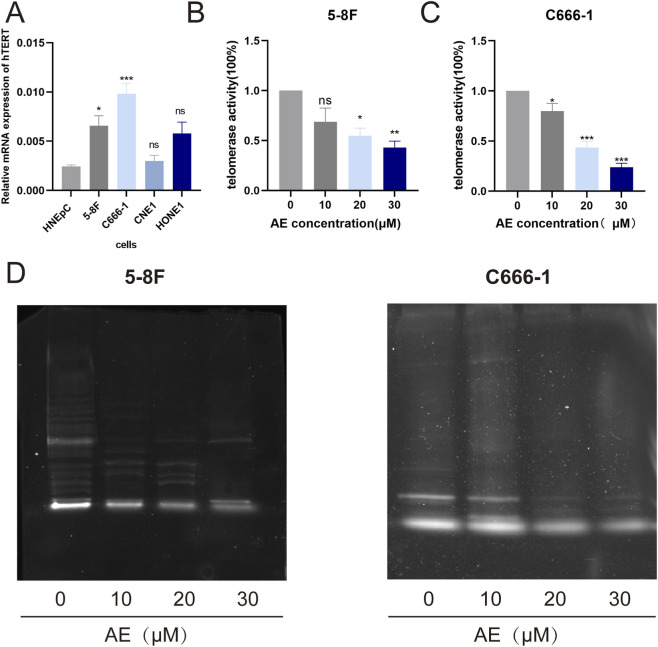
Expression of hTERT in nasopharyngeal carcinoma cell lines and inhibitory effect of AE on telomerase activity. **(A)** Relative expression levels of hTERT mRNA in normal nasal mucosal epithelial cells (HNEpC) and various nasopharyngeal carcinoma cell lines (CNE1, 5–8F, HONE1, C666-1) detected by real-time quantitative PCR. Compared with normal cells, hTERT was significantly upregulated in 5–8F and C666-1 cells (*P < 0.05, **P < 0.01), whereas no significant difference was observed in CNE1 and HONE1 cells (^ns^ P > 0.05). **(B,C)** 5–8F and C666-1 cells were treated with different concentrations of AE for 48 h, and telomerase activity was detected by TRAP-qPCR. The results showed that AE inhibited telomerase activity in a dose-dependent manner. **(D)** TRAP-qPCR products were verified by polyacrylamide gel electrophoresis (PAGE), further confirming the inhibitory effect of AE on telomerase activity. Data are presented as mean ± standard deviation, n = 3. ^ns^P > 0.05, *P < 0.05, **P < 0.01, ***P < 0.001, compared with the control group.

To validate hTERT expression in nasopharyngeal carcinoma (NPC), qPCR was performed in normal nasal epithelial cells (HNEpC) and NPC cell lines (CNE1, HONE1, 5–8F, C666-1). Compared with HNEpC, hTERT expression was significantly upregulated in 5–8F and C666-1 cells (P < 0.05), whereas no significant difference was observed in CNE1 or HONE1 cells (Figure 1A). Therefore, 5–8F and C666-1 were selected for subsequent experiments. These results confirm that hTERT is highly expressed in both HNSC tissues and NPC cell lines, with a distinct and favorable prognostic association in HNSC.

### 3.2 AE inhibits telomerase activity and downregulates hTERT in a dose-dependent manner

**FIGURE 2:**
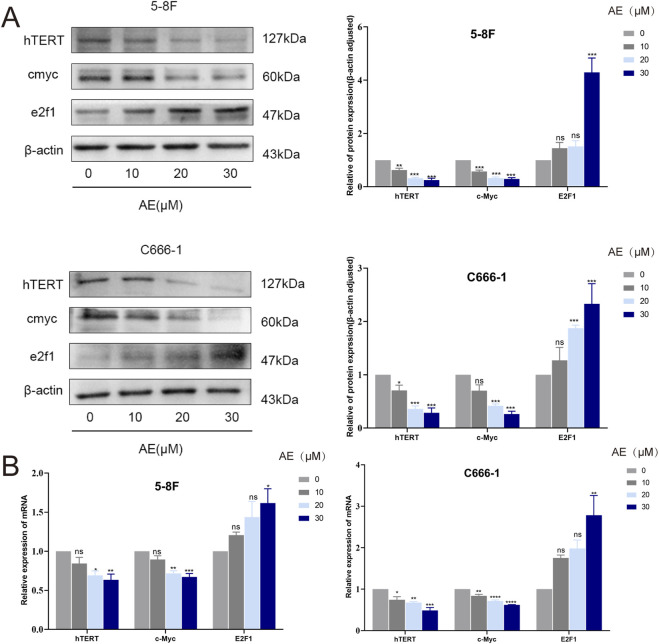
AE regulates hTERT expression and its upstream transcription factors c-Myc and E2F1 in NPC cells in a dose-dependent manner. 5–8F and C666-1 cells were treated with AE at concentrations of 0, 10, 20, and 30 μM for 48 h. **(A)** Protein expression levels of hTERT, c-Myc, and E2F1 were detected by Western blotting. β-actin served as the loading control. Representative blots are shown, with densitometric quantification presented as fold change relative to the control group. **(B)** Relative mRNA expression levels of hTERT, c-Myc, and E2F1 were detected by qRT-PCR, normalized to β-actin. All data are presented as mean ± SD, n = 3. ^ns^P > 0.05, *P < 0.05, **P < 0.01, ***P < 0.001 compared to the control group (0 μM).

Molecular docking predicted a potential interaction between AE and the active pocket of hTERT (Supplementary Figure S5). The effect of AE on telomerase activity was evaluated using TRAP-qPCR and PAGE. In both 5–8F and C666-1 cells, increasing concentrations of AE (0, 10, 20, 30 μM) for 48 h led to a significant, dose-dependent reduction in telomerase activity. This was shown by quantitative TRAP-qPCR and by the progressive weakening of telomeric ladder bands (Figures 1B–D). qPCR and Western blot analyses also showed that AE downregulated hTERT at both mRNA and protein levels in a dose-dependent manner (Figure 2). AE treatment also decreased c-Myc expression and increased E2F1 expression, both in a dose-dependent manner (Figure 2). These findings indicate that AE simultaneously downregulates c-Myc and upregulates E2F1. Notably, the inverse correlation between E2F1 and hTERT upon AE treatment is of particular interest, as previous studies have reported that E2F1 can repress hTERT transcription in certain cell types under genotoxic stress (Won et al., 2004; Crowe et al., 2001). However, whether this repressive mechanism operates in our NPC model requires direct experimental validation.

### 3.3 c-Myc overexpression partially rescues AE-Induced cell viability inhibition

**FIGURE 3:**
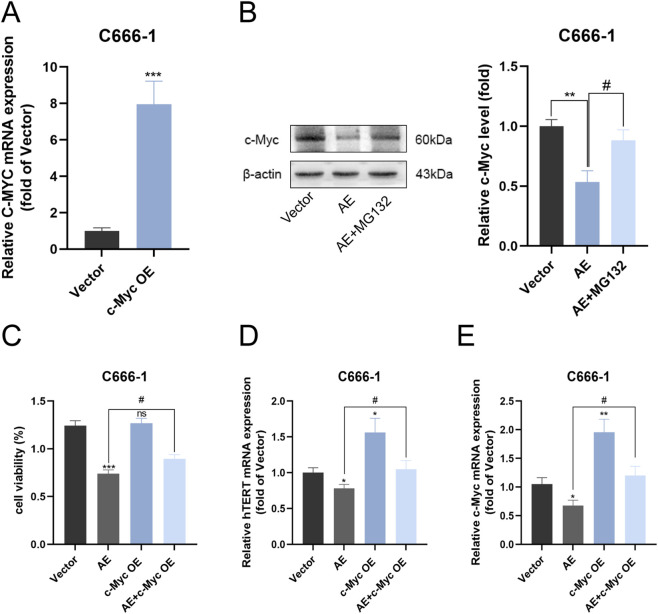
AE suppresses NPC cell viability via the c-Myc/hTERT axis. C666-1 cells were transfected with empty vector or c-Myc overexpression plasmid (c-Myc OE), followed by 48 h treatment with 20 μM AE or DMSO. **(A)** qPCR verification of c-Myc overexpression efficiency. c-Myc mRNA normalized to vector group; ***P < 0.001 vs. vector. **(B)** Western blot demonstrating AE triggers proteasome-dependent c-Myc degradation. Cells were incubated with 20 μM AE for 48 h, with 10 μM MG132 supplemented in the final 6 h β-actin served as internal reference; **P < 0.01 vs. vector, *P < 0.05 vs. AE. **(C)** CCK-8 cell viability rescue assay. AE significantly reduced cell viability (***P < 0.001 vs. vector). Single c-Myc OE exerted no significant impact (ns vs. vector), whereas c-Myc overexpression partially rescued AE-mediated growth suppression (^#^P < 0.05 vs. AE). **(D)** qPCR detection of hTERT mRNA levels. AE downregulated hTERT transcription (*P < 0.05 vs. vector). c-Myc OE significantly elevated hTERT (*P < 0.05 vs. vector), and co-transfection restored hTERT expression (^#^P < 0.05 vs. AE). **(E)** qPCR detection of endogenous c-Myc mRNA. AE decreased basal c-Myc mRNA (*P < 0.05 vs. vector); c-Myc OE markedly upregulated c-Myc transcripts (**P < 0.01 vs. vector), and ectopic c-Myc expression partially reversed AE-induced c-Myc repression (^#^P < 0.05 vs. AE). All data are presented as mean ± SD, n = 3. ns, P > 0.05; *P < 0.05, **P < 0.01, ***P < 0.001 versus vector group; ^#^P < 0.05 versus AE single treatment group.

To verify the functional relevance of c-Myc in AE-mediated anti-NPC effects, rescue experiments were performed by overexpressing c-Myc in C666-1 cells. CCK-8 assays showed that AE significantly suppressed cell viability, while c-Myc overexpression alone had no significant effect. Importantly, c-Myc overexpression partially but significantly reversed AE-induced viability inhibition (Figure 3C). Mechanistically, AE treatment markedly decreased c-Myc protein expression, which was restored by co-treatment with the proteasome inhibitor MG132, indicating that AE promotes c-Myc degradation through the ubiquitin-proteasome pathway (Figure 3B). At the transcriptional level, qPCR analysis revealed that AE moderately suppressed c-Myc and hTERT mRNA expression. Conversely, c-Myc overexpression significantly upregulated both transcripts and partially restored their levels upon AE treatment (Figures 3D,E). These results further support that AE modulates the c-Myc/hTERT axis through transcriptional suppression and proteasome-mediated degradation of c-Myc.

### 3.4 Knockdown of hTERT synergizes with AE to suppress malignant behaviors of NPC cells

**FIGURE 4:**
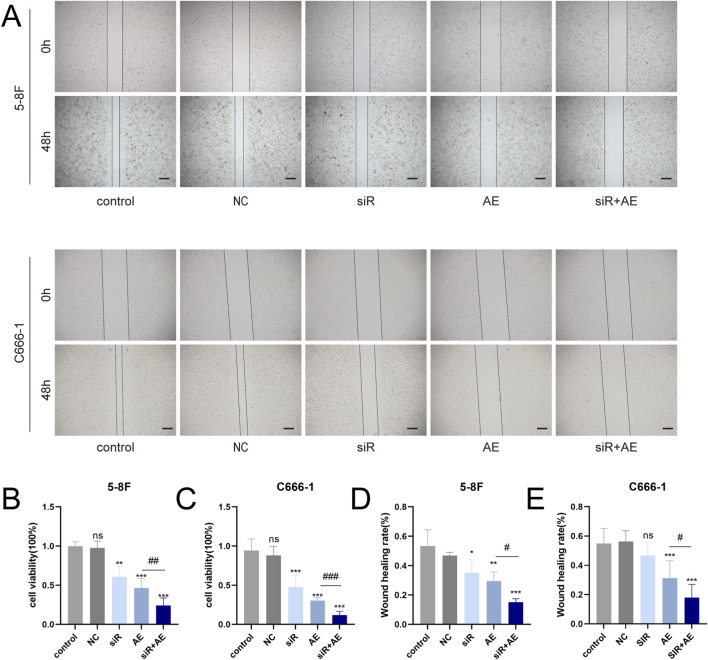
Effects of hTERT knockdown on the viability and migration of nasopharyngeal carcinoma cells. Cells were treated with five experimental conditions for 48 h: Control (untreated), NC (negative control siRNA), si-hTERT (hTERT knockdown), AE (20 μM), and si-hTERT + AE (combination). **(A)** Representative images of wound healing assay at 0 h and 48 h (scale bar = 100 μm). **(B)** Cell viability in 5–8F cells measured by CCK-8 assay. **(C)** Cell viability in C666-1 cells measured by CCK-8 assay. **(D)** Quantification of wound closure rates in 5–8F cells. **(E)** Quantification of wound closure rates in C666-1 cells. Data are presented as mean ± SD, n = 3. ^ns^P > 0.05, *P < 0.05, **P < 0.01, ***P < 0.001 vs. Control group; ^#^P < 0.05, ^##^P < 0.01 vs. AE group.

**FIGURE 5:**
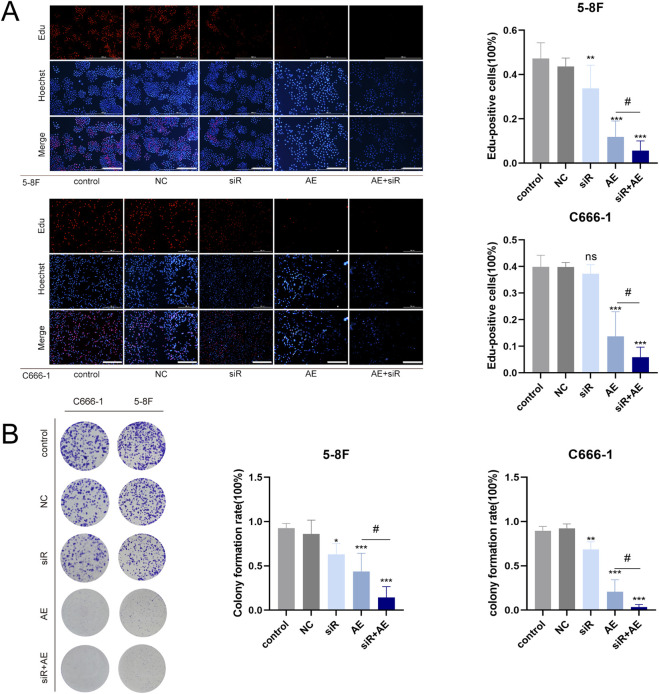
Knockdown of hTERT enhances the inhibitory effect of AE on NPC cell proliferation. 5–8F and C666-1 cells were treated with Control, NC (negative control siRNA), si-hTERT (hTERT knockdown), AE (20 μM), or si-hTERT + AE for 48 h. **(A)** Cell proliferation was assessed by EdU incorporation assay. Representative fluorescence images show EdU-positive cells (red) and Hoechst 33342-stained nuclei (blue). Scale bar = 200 μm. Data are presented as mean ± SD, n = 3. **(B)** Long-term clonogenic capacity was evaluated by colony formation assay. Representative images of crystal violet-stained colonies are shown, with quantification of colony numbers presented. Data are presented as mean ± SD, n = 3. ^ns^P > 0.05, *P < 0.05, **P < 0.01, ***P < 0.001 vs. Control group; ^#^P < 0.05 vs. AE group.

**FIGURE 6:**
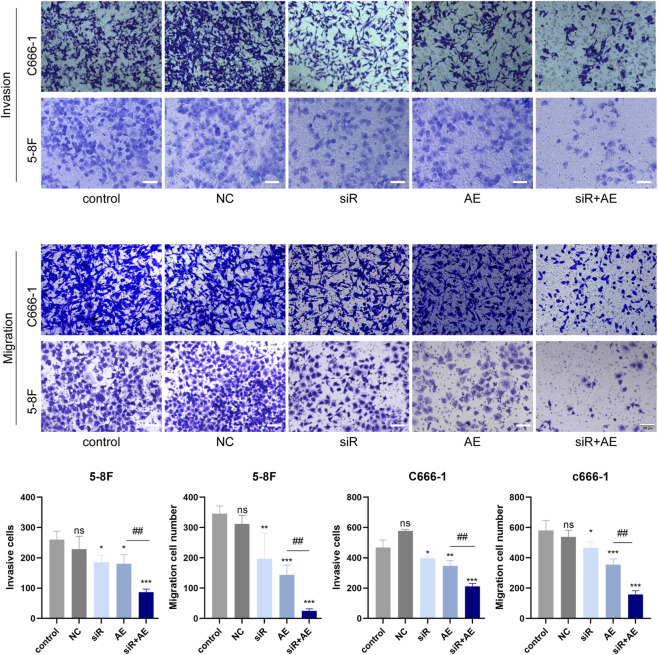
Knockdown of hTERT enhances the inhibitory effect of AE on NPC cell migration and invasion. 5–8F and C666-1 cells were treated with Control, NC (negative control siRNA), si-hTERT (hTERT k1nockdown), AE (20 μM), or si-hTERT + AE for 48 h. Cell migration and invasion were assessed using Transwell assays. For the invasion assay, the upper chambers were pre-coated with Matrigel; for the migration assay, uncoated chambers were used. Representative images of migrated and invaded cells stained with crystal violet are shown (scale bar = 100 μm). Quantification of migrated and invaded cell numbers is presented as mean ± SD, n = 3. ^ns^P > 0.05, *P < 0.05, **P < 0.01, ***P < 0.001 vs. Control group; ^#^P < 0.05, ^##^P < 0.01 vs. AE group.

**FIGURE 7:**
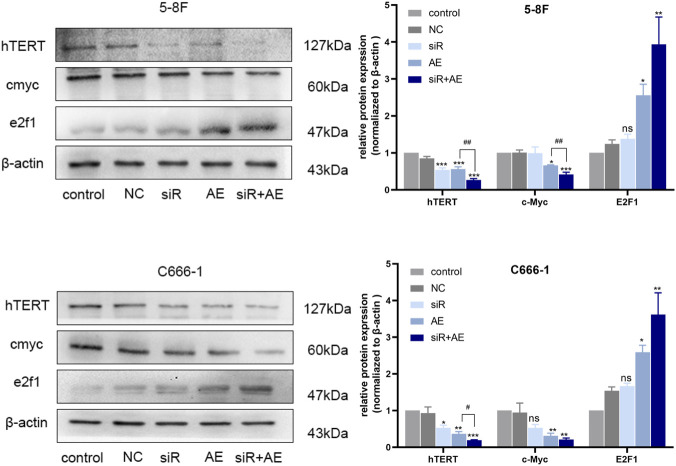
Knockdown of hTERT enhances the regulatory effects of AE on hTERT, c-Myc, and E2F1 protein expression. 5–8F and C666-1 cells were treated with Control, NC (negative control siRNA), si-hTERT (hTERT knockdown), AE (20 μM), or si-hTERT + AE for 48 h. Protein expression levels of hTERT, c-Myc, and E2F1 were analyzed by Western blotting. β-actin served as the loading control. Representative blots are shown, with densitometric quantification presented as fold change relative to the control group. Data are presented as mean ± SD, n = 3. ^ns^P > 0.05, *P < 0.05, **P < 0.01, ***P < 0.001 vs. control group; ^#^P < 0.05, ^##^P < 0.01 vs. AE group.

Having established c-Myc as a downstream mediator of AE, we next investigated whether hTERT silencing could synergize with AE to suppress the malignant phenotypes of NPC cells. The knockdown efficiency was confirmed by qPCR and FAM-labeled siRNA transfection assay (Supplementary Figure S6). In 5–8F cells, the CI value of the combination treatment was 0.468; in C666-1 cells, the CI value was 0.241. Both values indicated strong synergism. Isobologram analysis further confirmed the synergistic effect, with combination points located below the additive line in both cell lines. CCK-8 assays showed that either hTERT silencing or AE treatment alone significantly reduced cell viability, and the combination (si-hTERT + AE) exerted the strongest inhibitory effect (Figures 4B,C). EdU incorporation and colony formation assays further confirmed that the combination most effectively suppressed proliferation (Figures 5A,B). Wound-healing assays demonstrated that the combination significantly enhanced inhibition of cell migration compared with either monotherapy (Figures 4A, D, E). Similarly, Transwell assays revealed that the combination markedly reduced cell migration and invasion (Figure 6). Western blot analysis further confirmed that hTERT knockdown enhanced AE-induced downregulation of hTERT and c-Myc protein levels, while E2F1 expression was upregulated, consistent with the observed phenotypic changes (Figure 7). These results indicate that hTERT silencing synergizes with AE to suppress NPC cell proliferation, migration, and invasion.

### 3.5 hTERT silencing exacerbates AE-Induced ROS elevation

**FIGURE 8:**
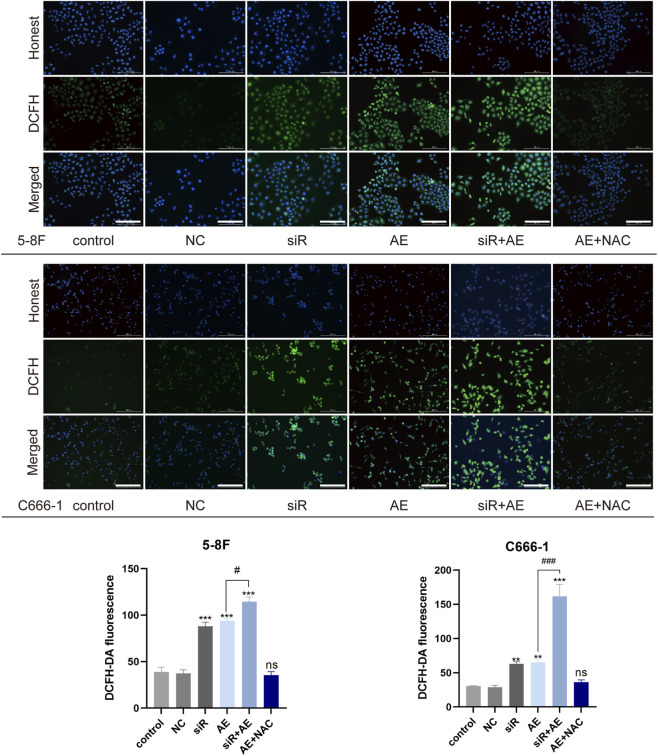
Knockdown of hTERT enhances AE-induced ROS accumulation in NPC cells. 5–8F and C666-1 cells were treated for 48 h under the following conditions: Control (untreated), NC (negative control siRNA), AE (20 μM), si-hTERT (hTERT knockdown), si-hTERT + AE (combination), and NAC + AE (pretreatment with 5 mM N-acetylcysteine for 2 h followed by 20 μM AE). Intracellular reactive oxygen species (ROS) levels were detected using the DCFH-DA fluorescent probe. Representative fluorescence images show DCFH-DA-derived fluorescence (green), with Hoechst 33342-stained nuclei (blue). Scale bar = 200 μm. Fluorescence intensity was quantified in at least 5 randomly selected fields per group. Data are presented as mean ± SD, n = 3. ^ns^P > 0.05, *P < 0.05, **P < 0.01, ***P < 0.001 vs. control group; ^#^P < 0.05, ^###^P < 0.001 vs. AE group.

To investigate the impact of hTERT knockdown on AE-induced oxidative stress, intracellular ROS levels were quantified using the DCFH-DA probe. AE treatment increased ROS accumulation in 5–8F and C666-1 cells, with this effect amplified by hTERT knockdown. Pretreatment with the antioxidant NAC completely inhibited AE-induced ROS production, establishing that ROS elevation was specific to the intervention (Figure 8). These results indicate that hTERT is involved in maintaining redox balance, and its absence increases NPC cell sensitivity to AE-induced oxidative stress.

### 3.6 hTERT silencing synergizes with AE to inhibit subcutaneous xenograft tumor growth in nude mice

**FIGURE 9:**
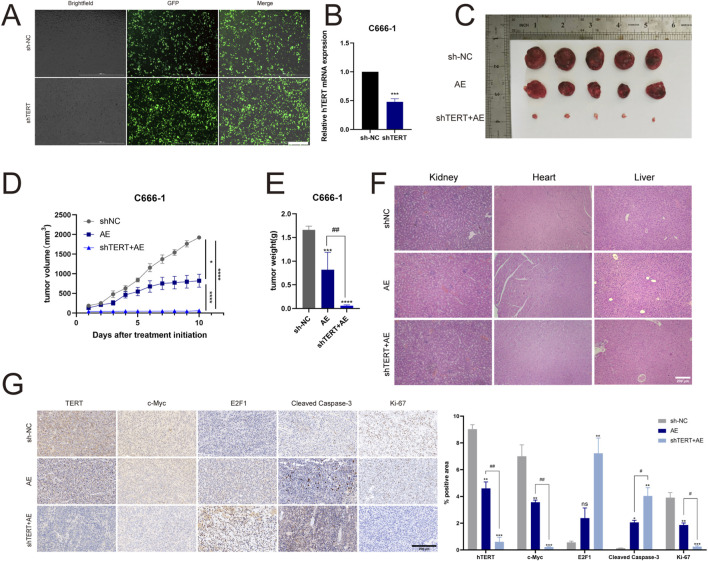
Stable hTERT knockdown synergizes with AE to inhibit NPC xenograft tumor growth *in vivo*. **(A,B)** Construction and validation of C666-1 cells with stable hTERT knockdown. C666-1 cells were infected with lentivirus carrying shRNA targeting hTERT (sh-hTERT) or negative control (sh-NC), selected with puromycin for 10–14 days. **(A)** Bright-field and GFP fluorescence images of infected cells (scale bar = 200 μm). **(B)** Knockdown efficiency of hTERT mRNA was verified by qPCR (n = 3). **(C–E)** Nude mice bearing subcutaneous xenografts were treated with daily intraperitoneal injections of AE (20 mg/kg). Groups: sh-NC (control), AE, and sh-hTERT + AE. **(C)** Representative images of dissected tumors. **(D)** Tumor growth curves showing tumor volume measured every 2–3 days. **(E)** Tumor weights at the end of the experiment. **(F)** Pathological analysis of major organs (heart, liver, kidney) from nude mice. Tissues were collected after treatment and stained with hematoxylin and eosin (H&E). No obvious histopathological changes were observed (scale bar = 200 μm; n = 5). **(G)** Immunohistochemical analysis of tumor tissues. Expression of hTERT, E2F1, c-Myc, Ki-67, and Cleaved Caspase-3 was detected by immunohistochemistry (scale bar = 200 μm). Data are presented as mean ± SD, n = 5. ^ns^P > 0.05, *P < 0.05, **P < 0.01, ***P < 0.001 vs. sh-NC group; ^#^P < 0.05, ^##^P < 0.01 vs. AE group.

To evaluate the *in vivo* synergistic effect, a subcutaneous xenograft model was established using C666-1 cells with stable hTERT knockdown (shTERT) or empty vector (sh-NC) (Figures 9A,B). Mice were treated with AE (20 mg/kg, i. p.) or vehicle daily for 10 days. Compared with the sh-NC + vehicle group, AE monotherapy significantly reduced tumor volume and weight. Importantly, the combination of shTERT and AE (shTERT + AE) further suppressed tumor growth, achieving the smallest tumor volume and weight among all groups (Figures 9C–E). H&E staining of heart, liver, and kidney tissues revealed no significant pathological changes in any group, indicating good *in vivo* safety of AE and shTERT lentivirus (Figure 9F). Immunohistochemical analysis of tumor tissues showed that the combination synergistically reduced hTERT and c-Myc expression, upregulated E2F1, decreased the proliferation marker Ki-67, and increased the apoptosis marker Cleaved Caspase-3 (Figure 9G). These findings suggest that hTERT silencing may synergize with AE to inhibit tumor growth and induce apoptosis by modulating the hTERT/c-Myc/E2F1 axis.

## 4 Discussion

**FIGURE 10:**
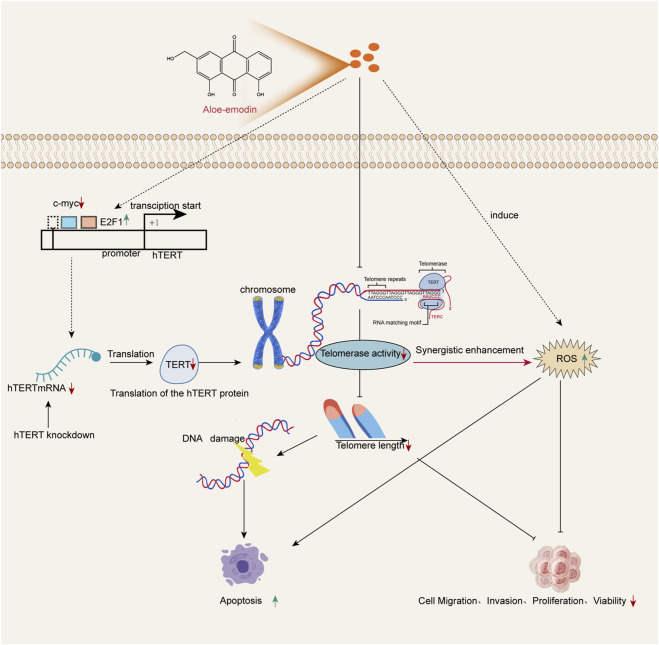
Graphical abstract. This schematic diagram summarizes the proposed anti-NPC effects of aloe-emodin (AE) observed in this study. AE treatment was associated with a reduction in hTERT expression and telomerase activity, together with modulation of the c-Myc/E2F1 signaling axis. Concurrently, AE combined with hTERT knockdown enhanced ROS induction and inhibited the malignant phenotypes of NPC cells. Dashed lines indicate proposed regulatory relationships that require further experimental validation. Created with BioGDP.com (Jiang et al., 2025).

This study provides evidence that in NPC, AE treatment is associated with suppression of hTERT expression and telomerase activity, together with differential regulation of its upstream transcription factors—downregulation of c-Myc and upregulation of E2F1. hTERT knockdown synergistically enhances the anti-proliferative, anti-migratory, and anti-invasive effects of AE, a mechanism associated with ROS accumulation and reinforced modulation of the c-Myc/E2F1/hTERT axis. *In vivo* xenograft models confirm the safety and efficacy of this combination strategy, providing experimental support for hTERT-targeted combination therapy in NPC (Figure 10).

### 4.1 hTERT as a distinctive prognostic and therapeutic target in NPC

A paradoxical prognostic association of hTERT was noted in HNSC, which may be explained by its reported immunomodulatory functions as a conserved self-tumor antigen (Xian et al., 2023; Mizukoshi and Kaneko, 2019). Importantly, this favorable prognostic pattern does not negate hTERT’s oncogenic role at the cellular level. Our qPCR validation confirmed hTERT overexpression in NPC cell lines (5–8F and C666-1), consistent with previous reports linking elevated hTERT mRNA to NPC clinical stage (Fu et al., 2017). Functional knockdown experiments showed that hTERT silencing suppressed proliferation, migration, and invasion, establishing hTERT as a driver of malignant phenotypes in NPC. Thus, hTERT shows dual clinical significance: it drives tumor aggressiveness at the cellular level and serves as a potential predictive biomarker for treatment response.

### 4.2 AE suppresses telomerase activity with concomitant modulation of c-myc and E2F1 expression

AE has been reported to exert anti-proliferative effects in various cancers (Jiang et al., 2021). Our study provides the first evidence in NPC that AE suppresses hTERT expression and telomerase activity. Functional assays confirmed that AE reduced telomerase activity and hTERT mRNA/protein levels in a dose-dependent manner.

The most intriguing finding lies in the differential regulation of hTERT’s transcription factors. AE downregulated c-Myc, a well-established activator of hTERT (Deng et al., 2018), consistent with reports that many natural products suppress hTERT via c-Myc inhibition (Qin et al., 2016). However, AE simultaneously upregulated E2F1. Although E2F1 can repress hTERT transcription in certain contexts (Won et al., 2004; Crowe et al., 2001), total E2F1 abundance does not necessarily reflect repressive activity, as its function is governed by phosphorylation status and promoter occupancy. In our model, AE-induced E2F1 upregulation was inversely correlated with hTERT expression, but whether this inverse relationship reflects a direct E2F1-mediated repression, a consequence of c-Myc downregulation, or an epiphenomenon of cellular stress cannot be distinguished from the current data. c-Myc downregulation likely plays the predominant role in hTERT suppression, given its well-established activating function and our rescue experiment results (Figure 3). A definitive assignment of E2F1’s role requires ChIP or knockdown-rescue experiments.

Whether AE directly binds to c-Myc, E2F1, or hTERT remains unknown. Molecular docking predicted a potential interaction with hTERT, but direct binding requires experimental confirmation (Wang et al., 2020). Nevertheless, our observations are clear: AE downregulates hTERT mRNA and alters c-Myc/E2F1 expression, indicating a transcriptional component. Our c-Myc overexpression experiments revealed that forced c-Myc expression only partially reversed the anti-proliferative effects of AE (Figure 3C). This partial rescue indicates that while c-Myc serves as a critical downstream mediator of AE in NPC cells, it is not the sole effector. AE, as a natural anthraquinone derivative, is inherently multifactorial. Beyond the c-Myc/hTERT axis, AE may concurrently engage other pathways, including the ROS-mediated stress response documented in this study (Figure 7) and other hTERT-regulating transcription factors such as SP1, HIF-1α, or NF-κB (Wu et al., 2021; Lu et al., 2021). This multitarget profile is consistent with the pharmacological characteristics of many plant-derived compounds and may contribute to AE’s therapeutic efficacy by imposing synergistic pressure on multiple oncogenic nodes. Thus, our data position c-Myc as an important—but not exclusive—downstream effector of AE in NPC.

### 4.3 hTERT knockdown synergizes with AE and exacerbates ROS accumulation

hTERT knockdown alone suppressed NPC malignant phenotypes. Combination with AE produced synergistic enhancement of these effects (CI < 0.9). Beyond its canonical telomere role, hTERT regulates redox homeostasis through non-canonical pathways (Ait-Aissa et al., 2022; Jayaprasad et al., 2024). In this study, hTERT knockdown exacerbated AE-induced ROS accumulation. This confirms that hTERT depletion sensitizes NPC cells to oxidative stress, while AE further stimulates ROS production—resulting in a “double-hit” effect.

Mechanistically, hTERT knockdown enhanced AE-induced downregulation of c-Myc. This suggests that loss of hTERT may influence the c-Myc/hTERT regulatory relationship. Elevated ROS suppresses c-Myc (Wu et al., 2018; Koo et al., 2023; Chen et al., 2022). In our model, hTERT knockdown increased basal ROS levels, and AE further elevated them, concomitant with decreased c-Myc expression. This raises the possibility of a self-reinforcing mechanism in which increased ROS downregulates c-Myc, potentially amplifying oxidative stress, although this hypothesis requires further investigation.

### 4.4 Limitations

First, the direct transcriptional regulation of hTERT by E2F1 was not verified by ChIP or promoter-reporter assays; thus, whether E2F1 upregulation directly represses hTERT or merely represents a stress-responsive epiphenomenon remains unclear. Second, the predicted AE–hTERT interaction lacks direct biophysical validation (e.g., SPR/ITC). Third, rescue experiments with E2F1 knockdown or hTERT overexpression were not performed, so the causal hierarchy along the c-Myc/E2F1/hTERT axis remains incompletely established. Fourth, contributions from other hTERT regulators (e.g., SP1, HIF-1α, NF-κB) cannot be excluded. Fifth, *in vivo* data are limited to a single cell line in a subcutaneous model, and long-term telomere dynamics were not assessed. Future studies addressing these gaps will further validate AE and hTERT-targeted strategies for NPC.

## Data Availability

The original contributions presented in the study are included in the article/Supplementary Material, further inquiries can be directed to the corresponding authors.

## Supplementary material

SUPPLEMENTARY FIGURE S1 hTERT is significantly upregulated in head and neck squamous cell carcinoma (HNSC) tumor tissues and is associated with better overall survival. **(A,B)** hTERT mRNA expression levels in HNSC tumor tissues compared with normal controls based on the ENCORI **(A)** and UALCAN **(B)** databases. ENCORI: tumor n = 520, normal n = 44; UALCAN: tumor n = 502, normal n = 44. Data are shown as log_2_(TPM+1). ***P < 1.0 × ×10^−12^ (ENCORI) and ***P < 1.3 × ×10^−17^ (UALCAN), Student’s *t*-test. **(C,D)** Kaplan-Meier overall survival curves of HNSC patients stratified by high vs. low hTERT expression using the GEPIA **(C)** and UALCAN **(D)** databases. High hTERT expression was associated with significantly longer overall survival (GEPIA: log-rank P = 0.034, HR = 0.75; UALCAN: P = 0.015). The median expression level was used as the cut-off. Data are presented as mean ± SD. *P < 0.05, **P < 0.01, ***P < 0.001. Abbreviations: HNSC, head and neck squamous cell carcinoma; TPM, transcripts per million; HR, hazard ratio.

SUPPLEMENTARY FIGURE S2 Differential expression of hTERT across pan-cancer. **(A)** TIMER2.0 (19 cancer types): hTERT was significantly upregulated in tumor tissues of 19 cancers, including HNSC, BLCA, BRCA, and CESC (*P < 0.05, **P < 0.01, ***P < 0.001). **(B)** Sangerbox (TCGA + +GTEx, 34 cancer types): hTERT was upregulated in 30 cancers (e.g., BLCA, HNSC) and downregulated only in TGCT (P < 0.05, **P < 0.01, ***P < 0.001). Data are shown as log2(TPM+1). Wilcoxon or t-test. Thus, hTERT is differentially expressed in 31 cancer types.

SUPPLEMENTARY FIGURE S3 Prognostic analysis of hTERT expression (TCGA pan-cancer, univariate Cox regression).High vs. low hTERT expression was associated with worse prognosis in multiple cancers (HR > 1, P < 0.05): **(A)** DFI: KIRP, KIPAN, THCA, ACC. **(B)** PFI: KIRP, KIPAN, GBMLGG, and others. **(C)** DSS: GBMLGG, KIRP, KIPAN, THCA. **(D)** OS: GBMLGG, KIPAN, KIRP, THCA.Thus, hTERT is an unfavorable prognostic factor in cancers such as KIRP, KIPAN, GBMLGG, and THCA.

SUPPLEMENTARY FIGURE S4 Correlation of hTERT expression with immune cell infiltration in HNSC.Using the TIMER2.0 and Sangerbox platforms, the relationship between hTERT expression and infiltration levels of six immune cell types (B cells, CD4^+^ T cells, CD8^+^ T cells, neutrophils, macrophages, and dendritic cells) was analyzed in head and neck squamous cell carcinoma (HNSC). Spearman’s rank correlation coefficients and P values are shown. A significant positive correlation was observed between hTERT expression and CD8^+^ T cell infiltration (Spearman’s ρ = 0.32, P < 0.001).

SUPPLEMENTARY FIGURE S5 Molecular docking of aloe-emodin (AE) with hTERT. Docking was performed using AutoDock Vina with the human hTERT cryo-EM structure (PDB ID: 9SI0). AE showed a strong predicted binding affinity of −10.5 kcal/mol and was positioned within the catalytic core region of hTERT. Specific interactions included hydrogen bonds with U-43 (RNA, 2.40 Å), Ser335 (2.10 Å), Tyr333 (3.30 Å), and Tyr562 (3.00 Å). The aromatic ring of AE also formed π–π stacking with surrounding tyrosine residues and hydrophobic interactions with nearby hydrophobic amino acids. These interactions suggest that AE binds preferentially within the protein catalytic pocket of hTERT, with additional contacts to the RNA template. This binding region is critical for hTERT reverse transcriptase activity; thus, AE may interfere with hTERT function through steric and molecular interactions. These *in silico* predictions provide a structural hypothesis for the inhibitory effects of AE on telomerase activity observed in this study.

SUPPLEMENTARY FIGURE S6 Validation of hTERT knockdown efficiency in nasopharyngeal carcinoma cells.5–-8F and C666-1 cells were divided into three groups: Control (untreated), empty vector control (siNC), and siRNA transfection group (si-hTERT). After 48 h of transfection, transfection efficiency was assessed using FAM-labeled siRNA, and hTERT mRNA expression was measured by real-time quantitative PCR. **(A)** FAM fluorescence images showed strong green fluorescence in the si-hTERT group, indicating successful siRNA delivery into cells (scale bar = 200 μm). **(B)** qPCR results demonstrated that hTERT mRNA expression was significantly reduced in the si-hTERT group compared with the Control and siNC groups (**P < 0.01), whereas no significant difference was observed between the siNC and Control groups (ns). Data are presented as mean ± SD, n = 3. *P < 0.05, **P < 0.01, ***P < 0.001 vs. Control group; ns indicates no statistical significance (P > 0.05).

SUPPLEMENTARY TABLE S1 Binding affinity values (Kcal/mol) for the top 9 conformations obtained from molecular docking between aloe-emodin and hTERT.

SUPPLEMENTARY TABLE S2 In GSE53819, the TERT probe (A_23_P110851) gave a log_2_FC of +0.136 (P = 0.567, adj.P = 0.704). In GSE12452, two independent probes for TERT (207199_at and 1555271_a_at) gave positive log_2_FC values of +0.064 and +0.019, respectively (adj.P > 0.05). No statistically significant differences were detected in any of the three analyses – likely due to the limited sample size (e.g., normal control n = 10 in GSE12452) and biological heterogeneity of clinical specimens.

SUPPLEMENTARY TABLE S3 Isobologram analysis and combination index (CI) calculation of the combined effect of AE and hTERT silencing in 5–-8F **(A)** and C666-1 **(B)** cells. CI values < 0.9 indicated significant synergistic effect. The combination data points located below the additive line confirmed strong synergism in both cell lines.
