# Supplementary material for: Aloe-emodin inhibits nasopharyngeal carcinoma by modulating telomerase activity involving the c-Myc/E2F1 axis
Source: Front Pharmacol. 2026 Jul 20;17:1850685. doi: 10.3389/fphar.2026.1850685 (PMC13429680; doi:10.3389/fphar.2026.1850685)
Supplement: Supplementary file 1 [file Table1.docx]

**Supplementary table 1. Molecular docking between Aloe-emodin and hTERT.**

| Rank | Affinity(Kcal/mol） |
| --- | --- |
| 1 | -10.5 |
| 2 | -10.1 |
| 3 | -10 |
| 4 | -9.9 |
| 5 | -9.7 |
| 6 | -9.7 |
| 7 | -9.7 |
| 8 | -9.6 |
| 9 | -9.6 |
