# Supplementary figures and images for: Aloe-emodin inhibits nasopharyngeal carcinoma by modulating telomerase activity involving the c-Myc/E2F1 axis
Source: Front Pharmacol. 2026 Jul 20;17:1850685. doi: 10.3389/fphar.2026.1850685 (PMC13429680; doi:10.3389/fphar.2026.1850685)

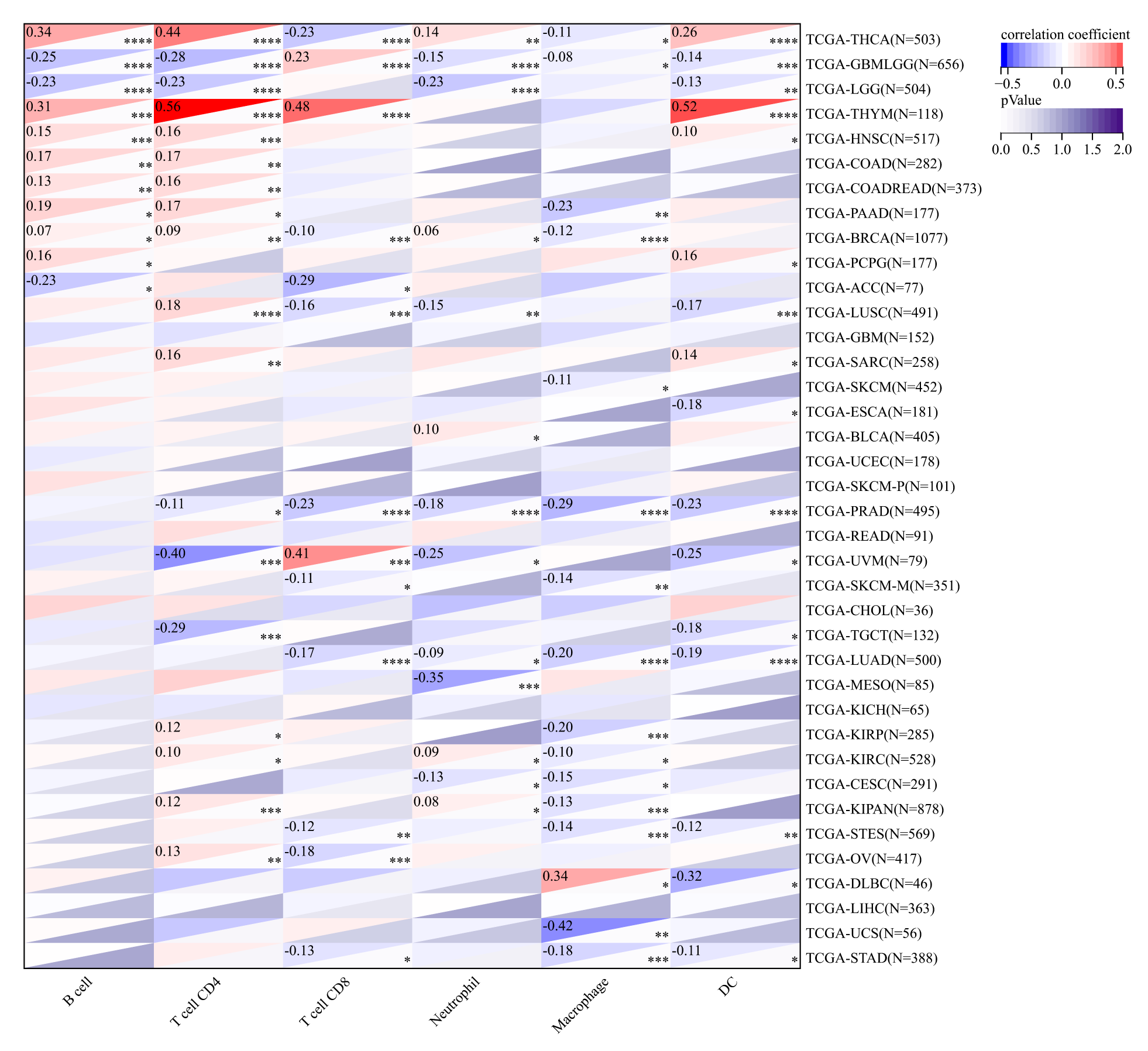

Supplement: Supplementary file 2 [file Image4.tif]

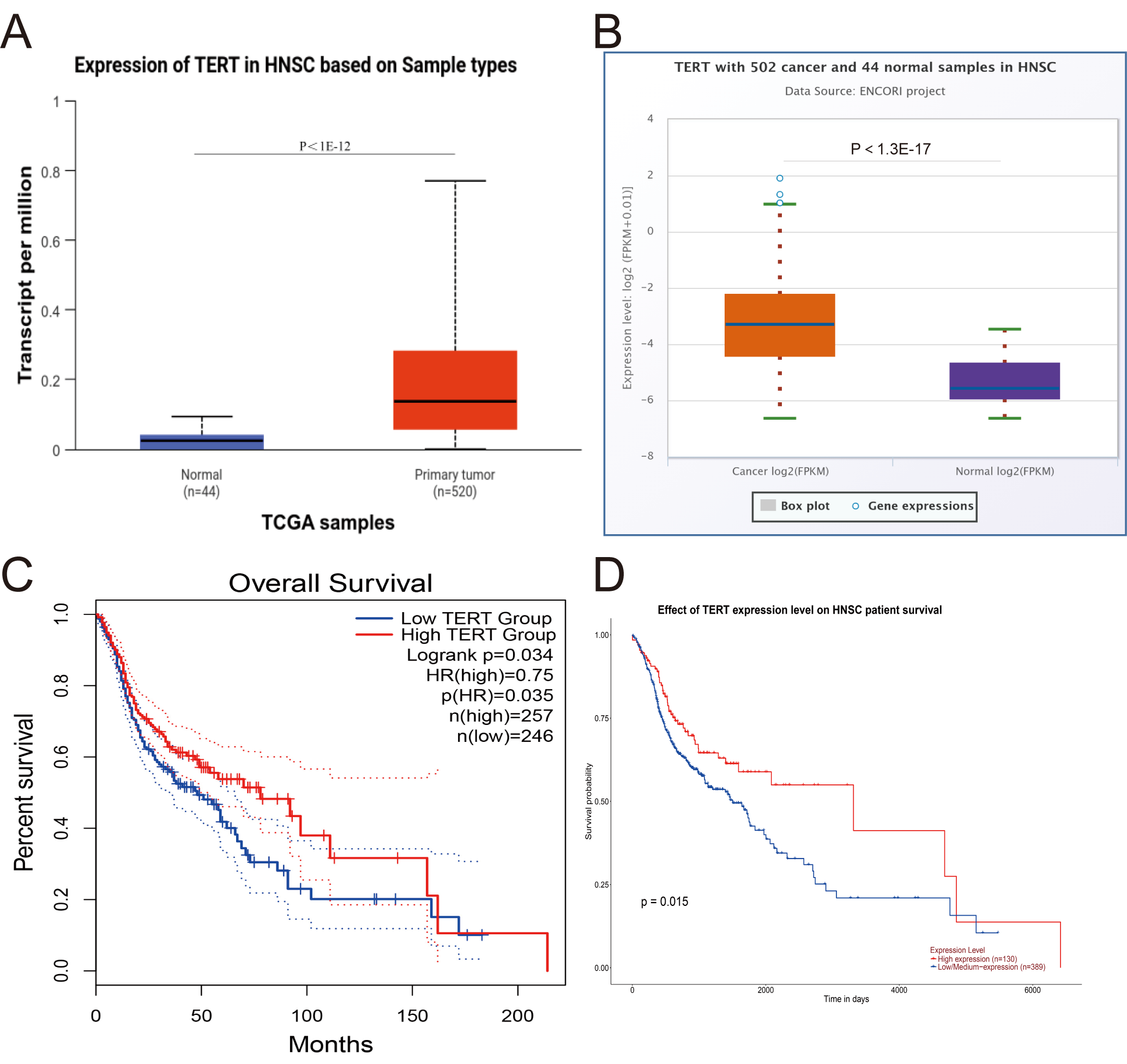

Supplement: Supplementary file 3 [file Image1.tif]

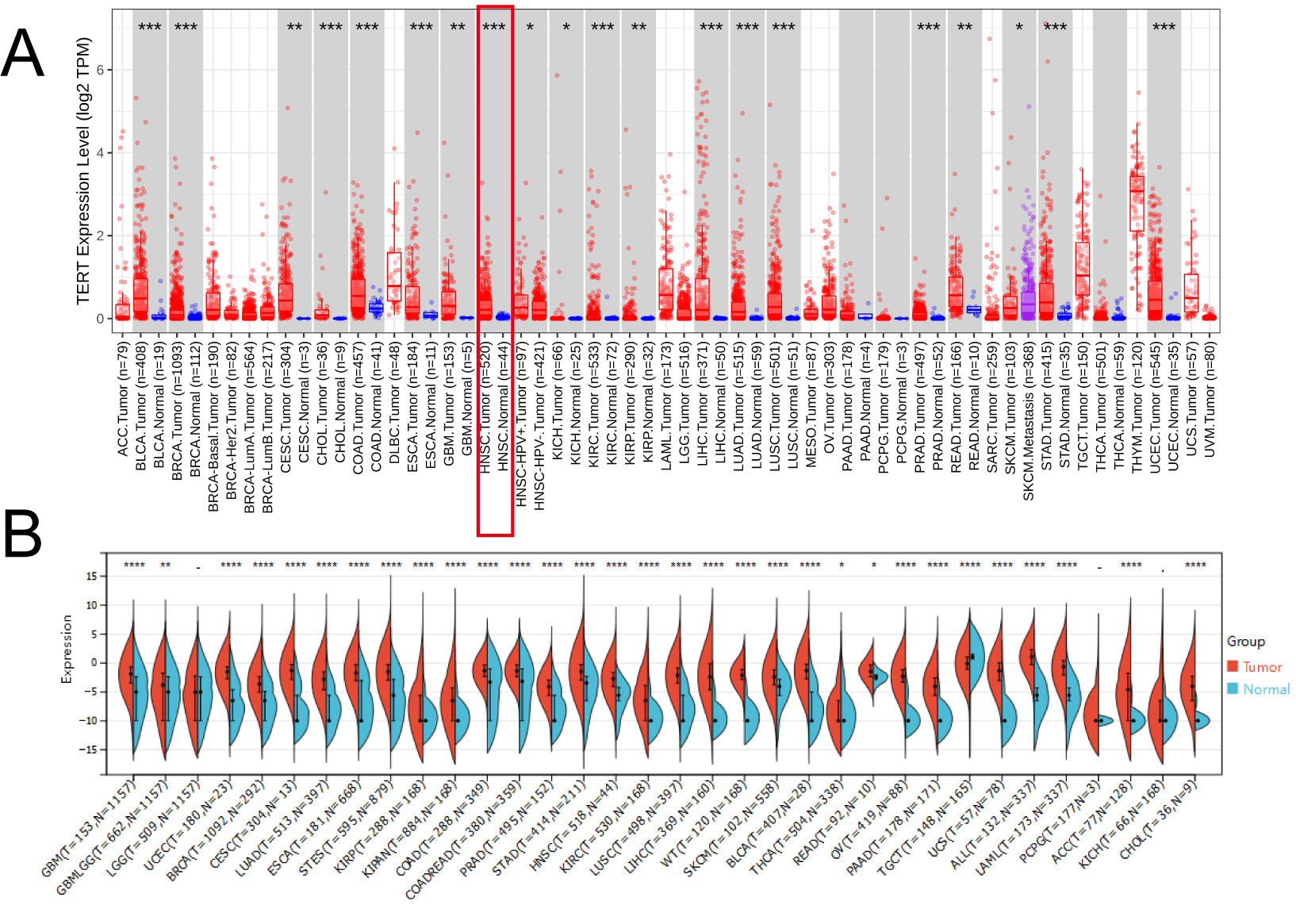

Supplement: Supplementary file 6 [file Image2.png]

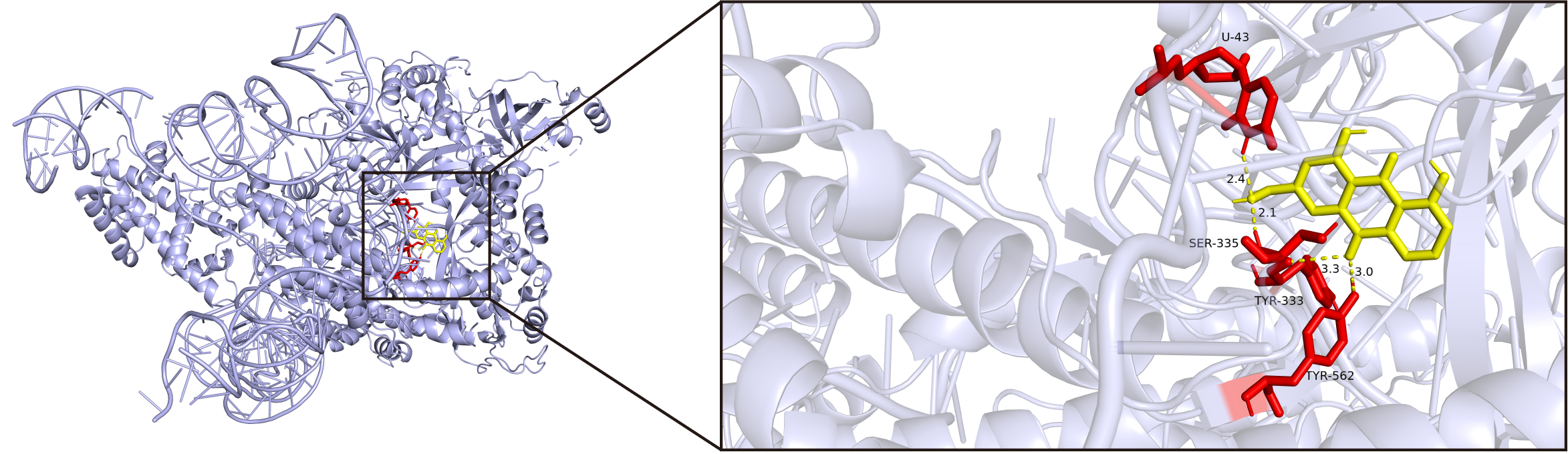

Supplement: Supplementary file 7 [file Image5.tif]

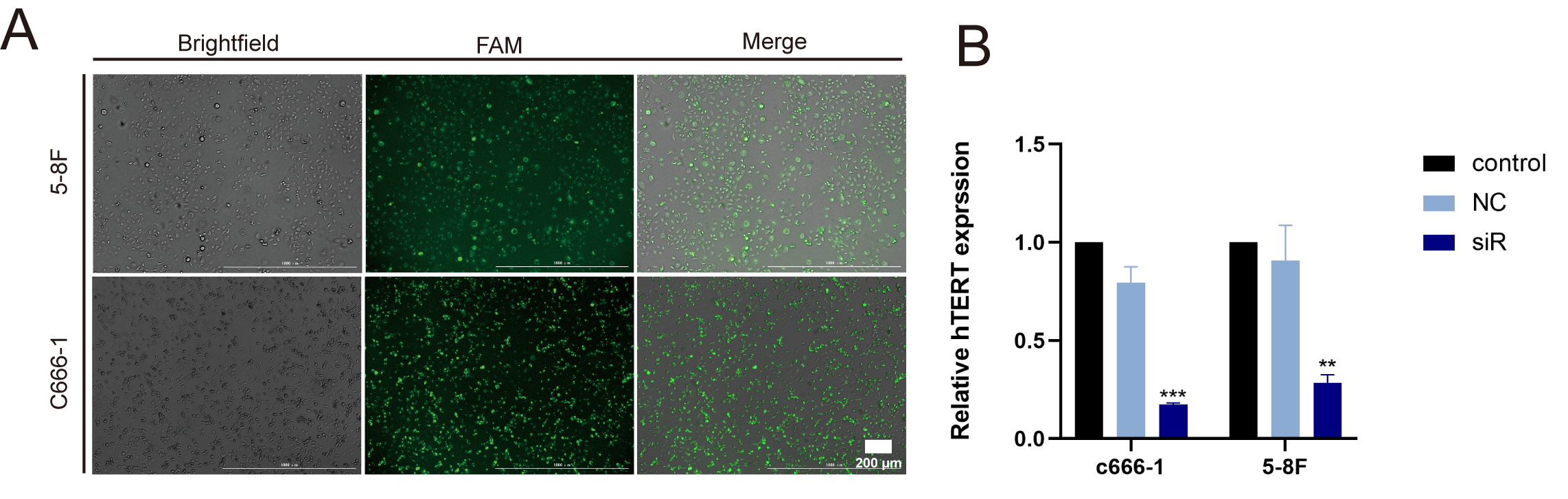

Supplement: Supplementary file 8 [file Image6.png]

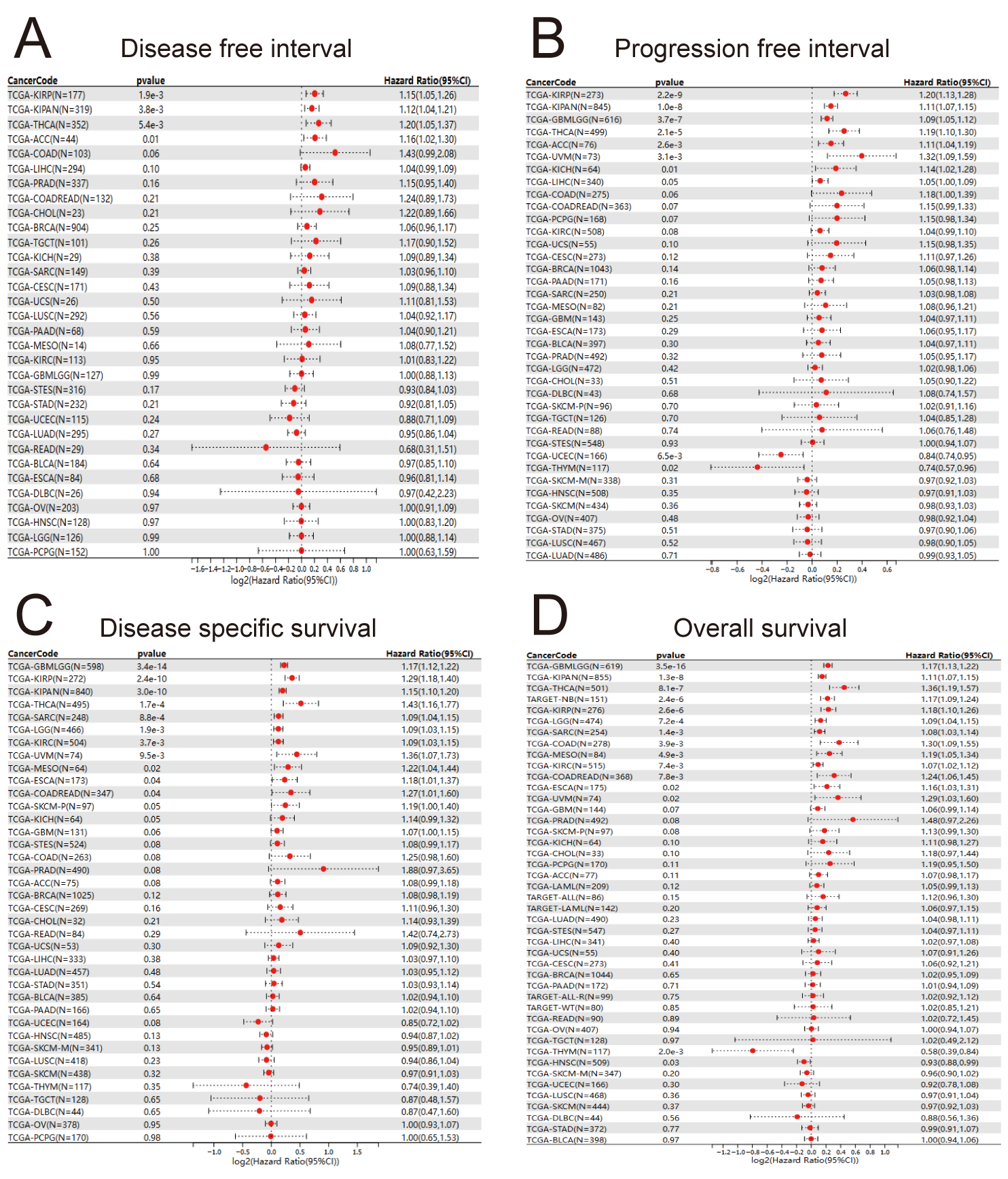

Supplement: Supplementary file 9 [file Image3.png]
