# Supplementary material for: Aloe-emodin inhibits nasopharyngeal carcinoma by modulating telomerase activity involving the c-Myc/E2F1 axis
Source: Front Pharmacol. 2026 Jul 20;17:1850685. doi: 10.3389/fphar.2026.1850685 (PMC13429680; doi:10.3389/fphar.2026.1850685)
Supplement: Supplementary file 4 [file Table2.docx]

**Supplementary table 2. Detailed information of GSE datasets.**

| GEO Dataset | Probe ID | log₂FC | P.Value | adj.P.Val | NPC (n) | Normal (n) |
| --- | --- | --- | --- | --- | --- | --- |
| GSE53819 | A_23_P110851 | +0.136 | 0.567 | 0.704 | 18 | 18 |
| GSE12452 | 207199_at | +0.064 | 0.436 | 0.642 | 31 | 10 |
| GSE12452 | 1555271_a_at | +0.019 | 0.829 | 0.912 | 31 | 10 |
