# Supplementary material for: Aloe-emodin inhibits nasopharyngeal carcinoma by modulating telomerase activity involving the c-Myc/E2F1 axis
Source: Front Pharmacol. 2026 Jul 20;17:1850685. doi: 10.3389/fphar.2026.1850685 (PMC13429680; doi:10.3389/fphar.2026.1850685)
Supplement: Supplementary file 5 [file Table3.docx]

**Supplementary table 3. Combination Index (CI).**

|  | 5-8F | C666-1 |
| --- | --- | --- |
| Effect | 75.8% | 85.9% |
| CI Value | 0.468 | 0.241 |
| Interpretation | Synergism | Synergism |
| Dose A (Combo) | 2.00e+1 | 2.00e+1 |
| Dose B (Combo) | 1.00e+0 | 1.00e+0 |
| DxA (Alone) | 7.83e+1 | 1.52e+2 |
| DxB (Alone) | 4.70e+0 | 9.14e+0 |
